# Intramuscular and intratendinous placenta‐derived mesenchymal stromal‐like cell treatment of a chronic quadriceps tendon rupture

**DOI:** 10.1002/jcsm.12894

**Published:** 2022-01-05

**Authors:** Tazio Maleitzke, Petra Reinke, Alison N. Agres, Sónia A. Alves, Levent Akyüz, Florian N. Fleckenstein, Anna Bichmann, Racheli Ofir, Carsten Perka, Georg N. Duda, Tobias Winkler

**Affiliations:** ^1^ Center for Musculoskeletal Surgery Charité – Universitätsmedizin Berlin, corporate member of Freie Universität Berlin and Humboldt‐Universität zu Berlin Berlin Germany; ^2^ Julius Wolff Institute Berlin Institute of Health at Charité – Universitätsmedizin Berlin Berlin Germany; ^3^ BIH Charité Clinician Scientist Program Berlin Institute of Health at Charité – Universitätsmedizin Berlin, BIH Biomedical Innovation Academy Berlin Germany; ^4^ Department of Nephrology Charité – Universitätsmedizin Berlin, corporate member of Freie Universität Berlin and Humboldt‐Universität zu Berlin Berlin Germany; ^5^ Berlin Institute of Health Center for Regenerative Therapies Berlin Institute of Health at Charité – Universitätsmedizin Berlin Berlin Germany; ^6^ Department of Diagnostic and Interventional Radiology Charité – Universitätsmedizin Berlin, corporate member of Freie Universität Berlin and Humboldt‐Universität zu Berlin Berlin Germany; ^7^ Department of Anesthesiology and Operative Intensive Care Medicine Charité – Universitätsmedizin Berlin, corporate member of Freie Universität Berlin and Humboldt‐Universität zu Berlin Berlin Germany; ^8^ Pluristem Therapeutics Inc. Haifa Israel

**Keywords:** Advanced therapies, Knee joint, Trauma, PLX‐PAD, Regeneration, Inflammation

## Abstract

**Background:**

Quadriceps tendon ruptures (QTRs) are rare but debilitating injuries, often associated with chronic metabolic conditions or long‐term steroid treatment. While the surgical treatment for acute QTRs is described thoroughly, no common strategy exists for the often frustrating treatment of chronic, reoccurring QTRs. The pro‐angiogenic and immunomodulatory properties of placenta‐derived adherent mesenchymal stromal‐like (PLX‐PAD) cells have been described to protect musculoskeletal tissues from inflammation and catabolic cytokine migration, yet little is known about the regenerative potential of PLX‐PAD cells in repetitively damaged tendon tissue.

**Case:**

We report the case of an 80‐year‐old male patient with a chronic three‐time QTR of his right knee. The quadriceps tendon was reconstructed applying a conventional suture anchor repair procedure combined with a synthetic mesh augmentation and additional intramuscular and intratendineous PLX‐PAD cell injections as an individualized treatment approach. No adverse events were reported, and excellent radiological and functional outcomes with a passive range of motion of 0/0/120° knee extension‐flexion were observed at the 12 month follow‐up. Gait analysis confirmed restoration of joint motion, including gait speed, deficit in step length, and knee extensor muscle strength (pre‐surgery: 0.98 m/s, 40 cm, 42.4 ± 12.4 N; 9 months post‐surgery: 1.07 m/s, 0 cm, 10.4 ± 18.9 N) as well as hyperextension throughout stance and late swing phases (pre‐surgery: −11.2 ± 0.9°; 9 months post‐surgery: −2.7 ± 1.6°). Postoperative lymphocyte and cytokine analyses from the patient's peripheral blood serum suggested a systemic short‐term immunoregulatory reaction with postoperatively increased interleukin (IL)‐6 (pre‐surgery: 0.79 pg/mL; day 1: 139.97 pg/mL; day 5: 5.58 pg/mL; 9 months: 1.76 pg/mL) and IL‐10 (pre‐surgery: 0.9 pg/mL; day 1: 1.21 pg/ mL; day 5: 0.3 pg/mL; 9 months: 0.34 pg/mL) levels that decreased again over time.

**Conclusions:**

Herein, we demonstrate a successfully treated chronic QTR with a synergistic surgical and biological reconstructive treatment approach. This local add‐on treatment with PLX‐PAD cells may be considered in specific cases of chronic QTRs, not susceptible to traditional suture anchor procedures and which exhibit a high risk of treatment failure. Further scientific engagement is warranted to explore underlying immunomodulatory mechanisms of action behind PLX‐PAD cell treatment for tendon injuries.

## Introduction

Quadriceps tendon ruptures (QTRs) are uncommon with an incidence of 1.37 in 100 000 per year and mostly occur due to eccentric loading to a partially flexed knee during a misstep or an attempt to regain balance. The injury is predominantly seen in men over 40 and often correlates with long‐term steroid intake or chronic conditions such as rheumatoid arthritis, renal failure, or diabetes mellitus.^1^ Typically, QTRs are associated with degenerative tendon changes^2^ and occur in the avascular region of the quadriceps tendon, located 1–2 cm above the superior patella margin.^3^ Immunoregulatory cytokines like interleukin (IL)‐1β, tumour necrosis factor (TNF)‐α, IL‐6, and vascular endothelial growth factor (VEGF) play a pivotal and complex role in the pathogenesis and healing of QTRs. While IL‐1β and TNF‐α are known to induce pro‐inflammatory mediators responsible for extracellular matrix degradation and suppression of type I collagen production, IL‐6 and VEGF support tendon healing through the expression of anti‐inflammatory IL‐10 and the induction of neo‐angiogenesis.^4^

The treatment of first‐time QTRs comprises early surgical reconstruction by suture anchor or transosseus repair techniques.^5^ No consensus has however been reached for when revision surgeries become necessary due to a chronic manifestation of reoccurring QTRs. Modified suture techniques,^6^, ^7^ allografts,^8^, ^9^ autografts,^10^ and synthetic mesh augmentations^11^, ^12^, ^13^ have been reported to provide additional mechanical stability in individual cases of chronic QTRs. However, so far none—to our knowledge—address the chronic inflammatory status.

Placenta‐derived adherent mesenchymal stromal‐like (PLX‐PAD) cells, derived from human donor placentas, show vast immunomodulatory and pro‐angiogenic capacities, including the inhibition of T‐cell proliferation and pro‐inflammatory cytokine release of TNF‐α and interferon (IFN)‐γ. They express typical mesenchymal stromal cell markers including CD13, CD73, CD90, and CD105 and are able to reduce oxidative stress, protect endothelial integrity, and increase capillary density and blood flow in pre‐clinical models of critical limb ischaemia.^14^, ^15^

**Figure 1:**
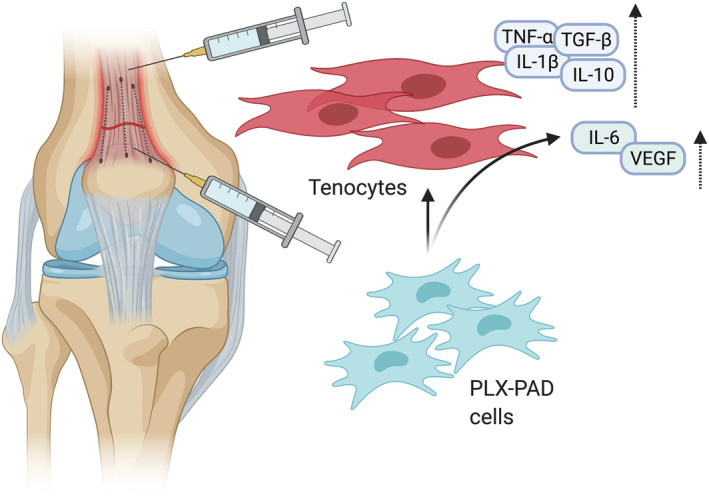
Schematic of a right knee joint with a reconstructed quadriceps tendon following a quadriceps tendon rupture and a proposed immunomodulatory mechanism of action of PLX‐PAD cell injections. Tenocytes respond with an enhanced production of tumor necrosis factor (TNF)‐α, transforming growth factor (TGF)‐β, interleukin (IL)‐1β, IL‐10, IL‐6, and vascular endothelial growth factor (VEGF) as a response to tendon injury. Placenta‐derived adherent mesenchymal stromal‐like (PLX‐PAD) cells enhance the increase of IL‐1β and IL‐6 in tenocytes and secrete IL‐6 and VEGF themselves when injected into the distal one‐fifth of the quadriceps muscle and the newly reconstructed quadriceps tendon. The figure was created with biorender.com.

We were previously able to demonstrate significantly improved gluteus muscle strength and volume following intramuscular injections of PLX‐PAD cells during hip arthroplasty.^16^ Further, PLX‐PAD cells were reported to secrete IL‐6 and VEGF when challenged with ischemic conditions *in vitro*.^17^ The cells have a favourable safety profile and are currently being investigated in two phase III trials for muscle regeneration (NCT03451916) and critical limb ischemia (NCT03006770). A first pre‐clinical animal study demonstrated an early inflammatory response combined with a transient beneficial effect on tendon failure load in a model of collagenase‐induced tendon degeneration treated with PLX‐PAD injections.^18^ The previous immunomodulatory and regenerative effects observed in various musculoskeletal tissues suggest that an intramuscular and intratendinous application of PLX‐PAD cells could improve and enhance tendon tissue integrity following injury (*Figure* *1*).

Herein, we report the case of an 80‐year‐old patient with a chronic third time QTR, who was successfully treated with local intramuscular and intratendinous injections of PLX‐PAD cells in addition to the surgical reconstruction of the chronically injured tendon. At the 12 month follow‐up, the patient was able to partake in an active lifestyle with an excellent functional outcome and a morphologically and radiologically intact quadriceps tendon. While clinically relevant changes in biomarker and gait analyses are described in respective chapters, no statistical evaluations were conducted due to the small sample size of *n* = 1.

## Case

We report the case of an 80‐year‐old male patient (body mass index of 28.7) who was transferred to our department from another clinic with the inability to extend the right knee after an accidental misstep upon descending stairs the same day.

After ruling out a fracture of the affected extremity, the patient transfer was initiated because of his past medical history of two previous QTRs on the now affected right side within two months, and three previous QTRs on the contralateral side within three months. The last QTR on both sides dated back seven months and in addition to the acutely ruptured quadriceps tendon on the right side, the left side also showed a partial suprapatellar gap, yet with remaining function to sufficiently stabilize the leg.

**Figure 2:**
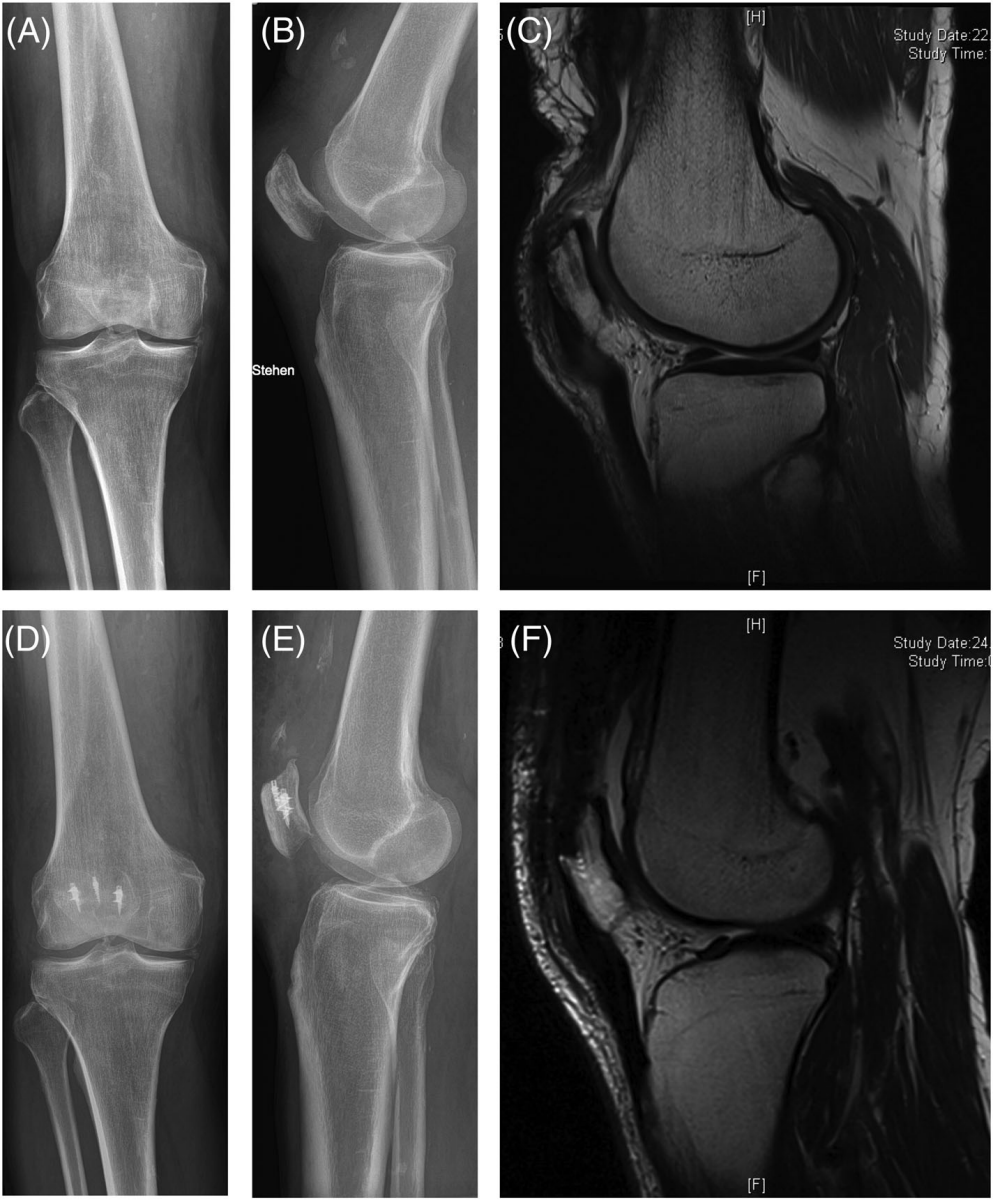
Radiographic images before and after quadriceps tendon reconstruction *(A)* Pre‐operative AP and *(B)* lateral X‐ray views of the right knee joint with a chronic quadriceps tendon rupture, visible in the *(C)* magnetic resonance imaging. *(D)* AP and *(E)* lateral X‐ray views of the reconstructed quadriceps tendon with titanium suture anchors placed in the patella. *(F)* The magnetic resonance imaging shows a reconstructed quadriceps tendon.

Clinical examination of the right knee revealed an old scar from previous surgeries and a complete suprapatellar gap accompanied by moderate periarticular swelling. X‐ray and magnetic resonance imaging confirmed the diagnosis of a re‐re‐QTR without further acute osseous or ligamentous (intact anterior and posterior cruciate ligament, lateral and medial collateral ligament and medial patellofemoral ligament) pathologies (*Figure* 2A–2C).

The previous five surgeries, which were undertaken elsewhere, comprised several suture anchor repair procedures. Comorbidities of the patient included coronary artery disease (including stent implantation), hypertension, hypolipoproteinaemia, chronic obstructive pulmonary disease, hypothyroidism, and a left nephrectomy.

As all previous surgical reconstructions had failed within rather short time frames, an individual treatment approach was agreed upon with the patient, which employed intramuscular and intratendineous PLX‐PAD cell injections as a compassionate use to facilitate a local immune‐modulation. The PLX‐PAD cell‐based therapy complemented a conventional suture anchor repair procedure combined with a synthetic mesh augmentation.

## Surgery

**Figure 3:**
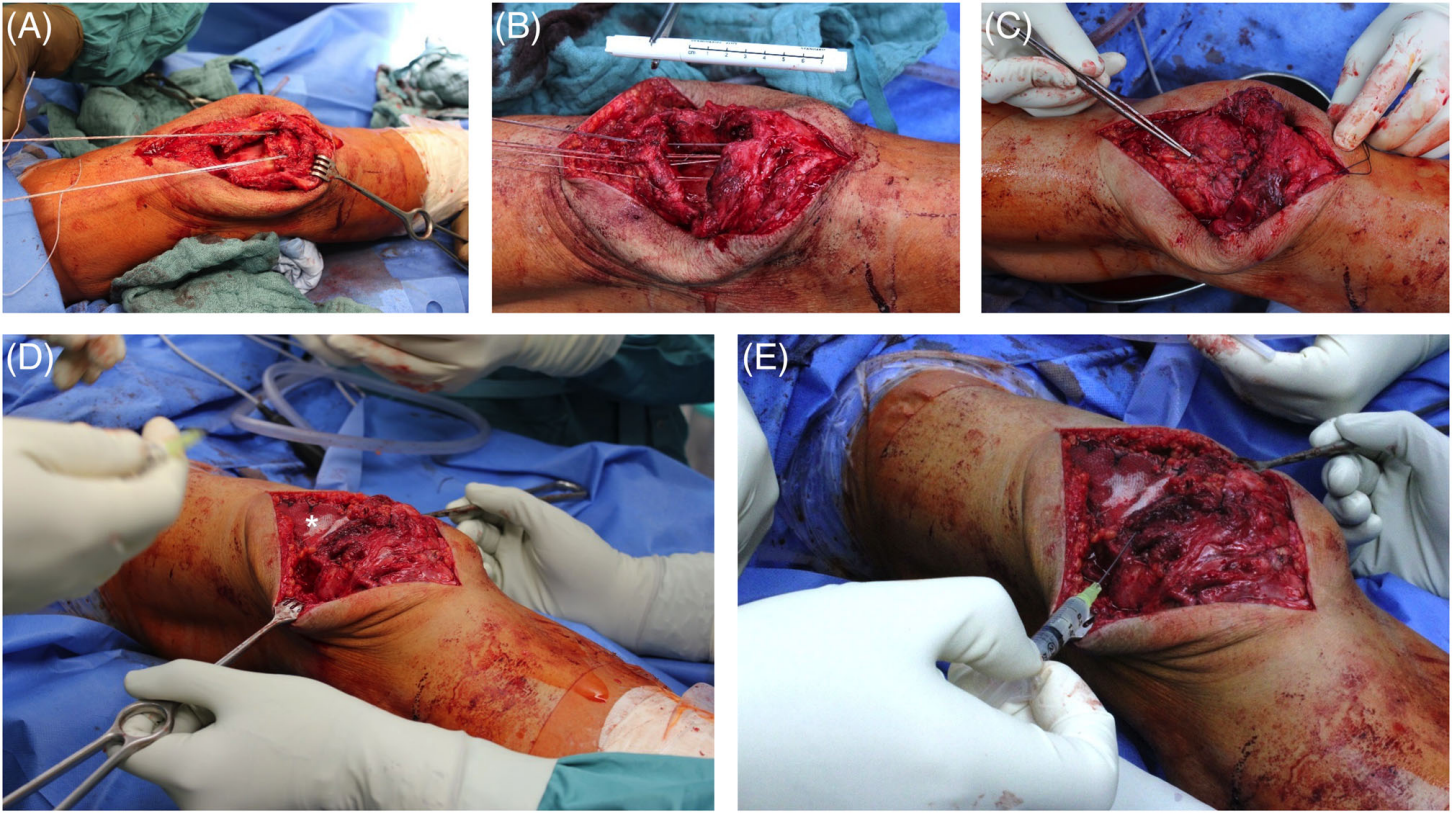
Chronologic intraoperative images of the surgical and biological quadriceps tendon reconstruction. *(A)* A defect measuring 7 × 5 cm can be observed resulting from the chronic QTR next to two titanium suture anchors placed in the patella. *(B)* FiberWire threads (Arthrex, Naples, FL, USA) attached to three titanium suture anchors aid the reattachment of the ruptured proximal tendon stump towards the patella. *(C)* After applying the Krackow suture technique, both tendon stumps were readapted. *(D)* A synthetic mesh graft (Bard® SoftMesh, Franklin Lakes, NJ, USA) (white asterisk) was used for augmentation. *(E)* At the end of the operation, PLX‐PAD cells were injected into the distal one‐fifth of the vastus lateralis and medialis muscle as well as the rectus femoris muscle of the quadriceps muscle and the newly reconstructed quadriceps tendon.

The patient was placed in a supine position, and a longitudinal midline incision was performed using the old scar, extending 5 cm cranial to the superior patella margin. The suprapatellar quadriceps tendon defect measured 7 cm in length and 5 cm in width (*Figure* 3A). The frayed and fibrotic ends were thoroughly debrided. Three titanium suture anchors were placed in the patella, and FiberWire threads (Arthrex, Naples, FL, USA) were placed in the quadriceps tendon using the Krackow technique (*Figure* 3B). An alignment of both ends was achieved (*Figure* 3C). To augment the repaired tendon, a synthetic polypropylene mesh (Bard® SoftMesh, Franklin Lakes, NJ, USA) was attached to the proximal patella tendon and the prepatellar fibrous tissue and sutured onto the distal quadriceps tendon (*Figure* 2D). Thawed PLX‐PAD cells were then injected into the distal one‐fifth of the vastus lateralis and medialis muscle as well as the rectus femoris muscle of the quadriceps muscle (75 × 10^6^ cells, 7.5 mL) and the quadriceps tendon itself (75 × 10^6^ cells, 7.5 mL) (*Figure* 2E). No adverse reactions were observed during or after the operation.

## Recovery

Postoperative management included 4 weeks of knee joint immobilization with 0° knee flexion and 15 kg partial weight bearing. This was followed by 2 weeks of 30° knee flexion and full weight bearing, followed by 2 weeks of 60° knee flexion and free range of motion (ROM) afterwards. Postoperative X‐ray and magnetic resonance imaging radiographs showed correctly placed titanium suture anchors in the patella as well as a fully restored quadriceps tendon (*Figure* 2D–2F).

The patient recovered and was assisted by physiotherapy to regain his pre‐operative ROM for the following 6 months. At the 12 month follow‐up, the patient's active and passive ROM was at 0/0/120° knee extension‐flexion. Further, the patient resumed participation in an elderly sports group 6 months after the operation without notable restrictions.

Of note, the left quadriceps tendon currently shows progressive signs of insufficiency, including a palpable median suprapatellar defect with remaining tendinous fascicles medially and laterally. Nonetheless, the patient's quadriceps muscle function is preserved, which is why we currently do not recommend further surgical interventions. However, both quadriceps tendons will be closely monitored during regular outpatient clinic visits.

## Gait analyses

The patient's pre‐operative and postoperative function at 3 and 9 month follow‐up visits was objectively evaluated in a dedicated motion capture laboratory (for details, please see Supporting Information, *Data* S1).

**Figure 4:**
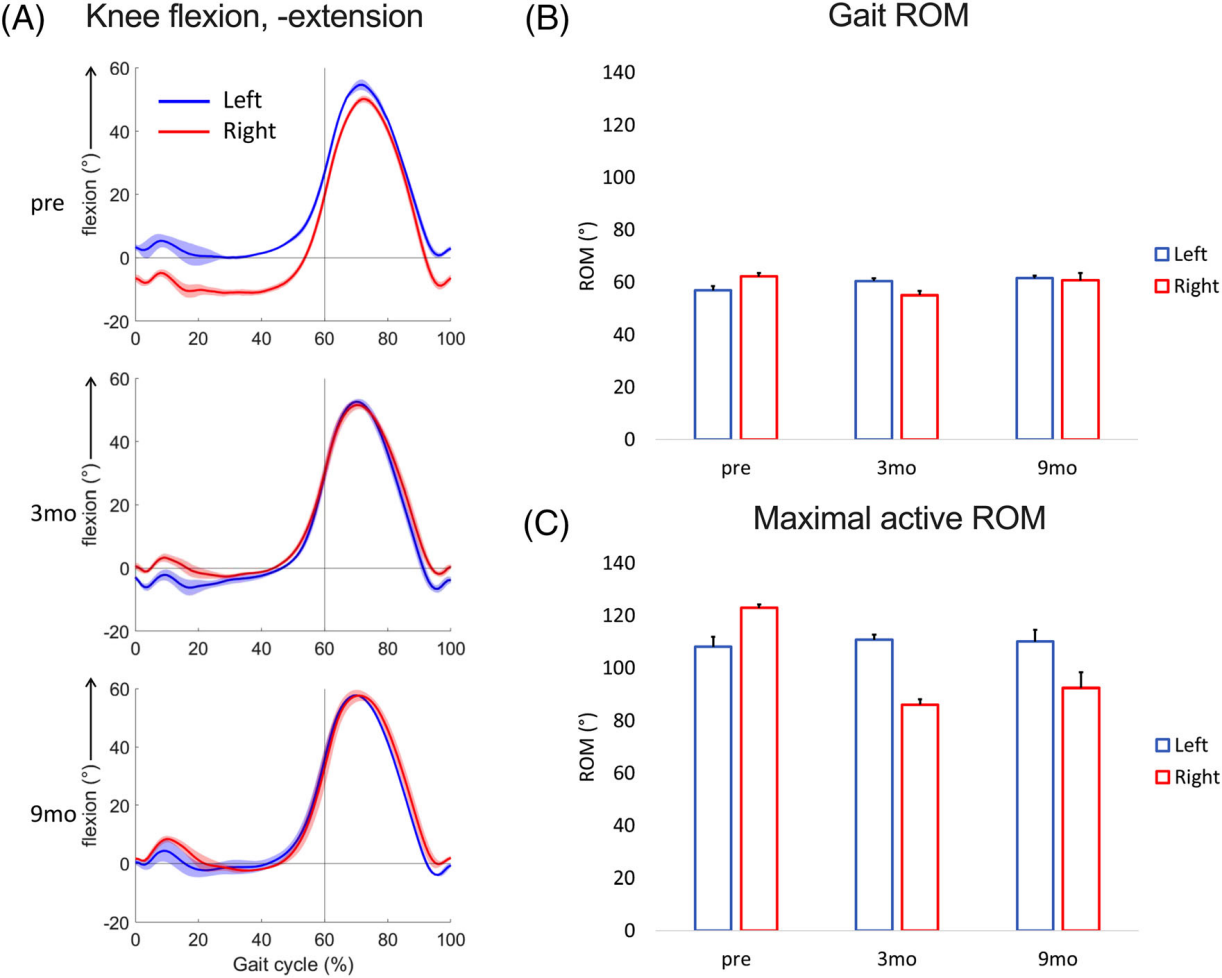
Gait analyses of the patient at three consecutive time points. *(A)* Mean curves (bold) with standard deviation (shaded) for knee flexion angles during gait. The stance and swing phases of the gait cycle are indicated to the left and right of the vertical line, respectively. *(B)* Knee range of motion (ROM) during gait and *(C)* maximal active knee flexion and extension. Blue and red colours indicate left and right side, respectively, plotted at pre‐operative (pre), 3 month (3mo), and 9 month (9mo) follow‐up visits.

Self‐selected gait speed, deficits in step length, and knee extensor muscle strength improved from pre‐surgery (0.98 m/s, 40 cm, 42.4 ± 12.4 N) at both 3 month (1.03 m/s, 10 cm, 31.2 ± 12.4 N) and 9 month (1.07 m/s, 0 cm, 10.4 ± 18.9 N) follow‐ups. Before surgery, the right knee exhibited moderate hyperextension (maximum −11.2 ± 0.9°) in stance and late swing phases, and the maximal knee flexion was lower on the right limb compared with the left (50.9 ± 1.1° vs. 55.6 ± 0.4°, *Figure* 4A). At both post‐surgical follow‐ups, hyperextension was reduced compared with pre‐operative levels (−3.1 ± 0.9° at 3 months; −2.7 ± 1.6° at 9 months) throughout stance and late swing phases (*Figure* 4A). Because of the pre‐surgical hyperextension, the right sagittal knee ROM was higher compared with the left during both gait and maximal knee flexion‐extension (*Figure* 4B and 4C, respectively). At the 3 month follow‐up, sagittal knee ROM during gait was lower on the right side compared with the left and at the 9 month follow‐up; no side‐to‐side differences remained. During gait, the patient tolerated only limited weight bearing at pre‐surgery (right: 91.0 ± 1.8 BW%, left: 88.5 ± 3.9 BW%) and 3 month post‐surgery (right: 96.4 ± 3.7 BW%, left: 94.0 ± 4.7 BW%), due to the use of walking aids. At the 9 month follow‐up, this was resolved (right: 101.8 ± 6.5 BW%, left: 112.0 ± 11.1 BW%).

## Biomarker analyses

**Figure 5:**
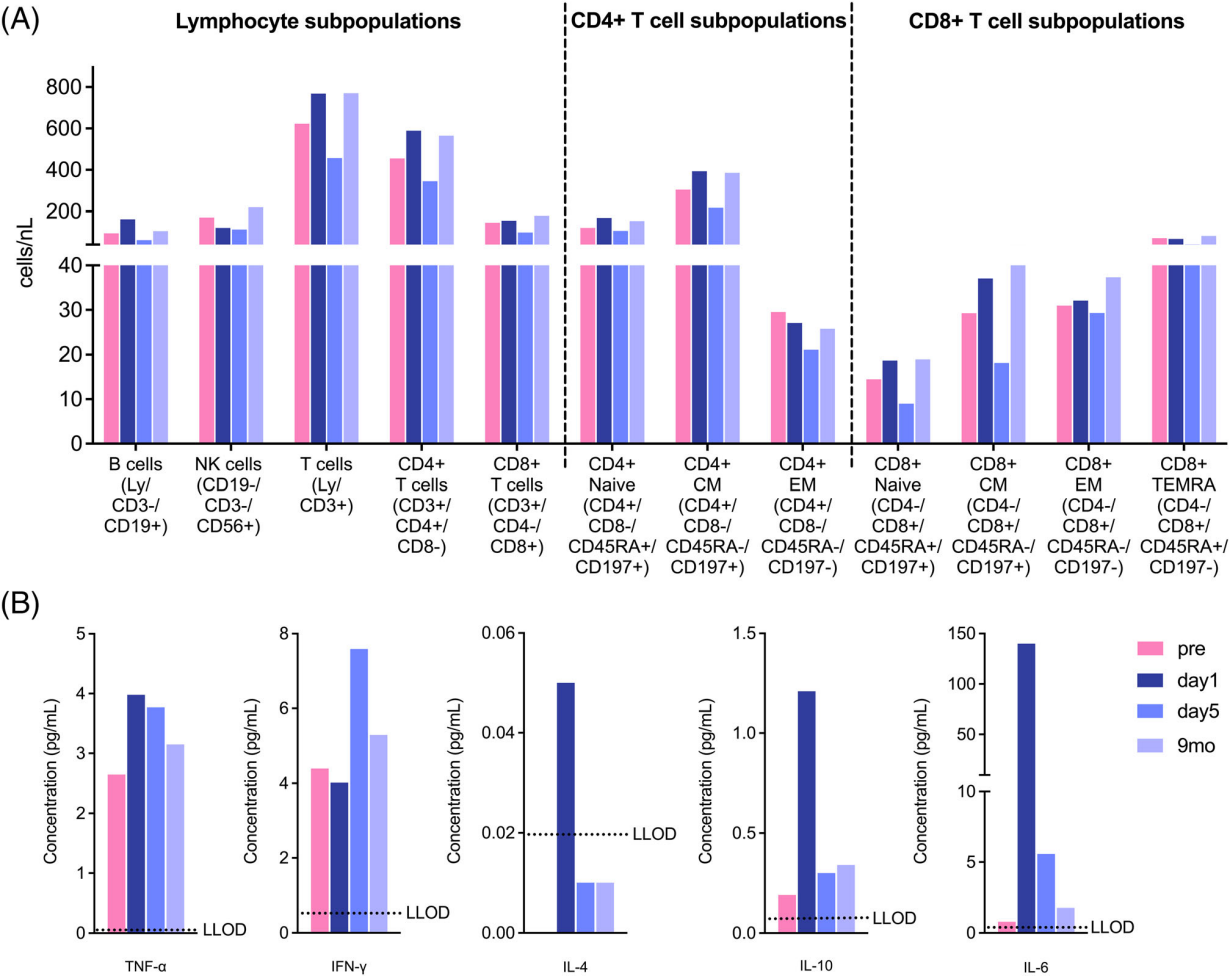
Flow cytometry data on lymphocytes and meso scale discovery data on pro‐inflammatory and anti‐inflammatory cytokines of the patient's peripheral blood serum at consecutive timepoints. *(A)* Cell count per nL whole blood at four different timepoints of B cells, natural killer (NK) cells, T cells, CD4+ cells, CD8+ cells, and CD4+ T cell subpopulations including CD4+ naive cells, CD4+ central memory (CM) cells, and CD4+ effector memory (EM) cells and CD8+ T cell subpopulations including CD8+ naive cells, CD8+ CM cells, CD8+ EM cells, and CD8+ terminally differentiated effector memory cells re‐expressing CD45RA (TEMRA) with the corresponding gating strategy in brackets. *(B)* Endogenous peripheral blood serum concentrations of tumor necrosis factor (TNF)‐α, interferon (IFN)‐γ, interleukin (IL)‐4, IL‐10, and IL‐6 in pg/mL at four different timepoints. The lower limit of detection (LLOD) (dotted line) reflects the sensitivity limit of the assay.

Pre‐surgical and post‐surgical lymphocyte and cytokine analyses of the patient's peripheral blood serum were conducted in order to identify potential immune alterations through flow cytometry and meso scale discovery technology (for details, please see Supporting Information, *Data* S2). Both B and T cells, including CD4+ helper cells and cytotoxic CD8+ cells increased on the first postoperative day, while natural killer (NK) cells decreased compared with pre‐operative values. A decrease was further seen for all aforementioned cells on the fifth postoperative day compared with day 1 and pre‐surgical values. After 9 months, both B cell and T cell subpopulations increased again and slightly overshot day 5 and pre‐operative levels (*Figure* 5A).

CD4+ T cell subpopulations showed an increase of naive and central memory (CM) cells, while effector memory (EM) cells decreased on day 1 compared with pre‐operative values.

CD8+ memory T cell subpopulations, including naive, CM and EM cells also increased on day 1 compared with pre‐operative numbers. Yet, on day 5, a decrease compared with day 1 and pre‐surgical values was observed, before all values slightly rose again above pre‐surgical and day 1 levels after 9 months. CD8+ terminally differentiated effector memory cells re‐expressing CD45RA (TEMRA) showed a decrease on day 5 and an increase again at the 9 month follow‐up which slightly overshot day 1 and pre‐operative values. For numeric values, please see *Table* 1.

**Table 1:** Numeric values of lymphocyte and cytokine concentrations pre‐surgery, 1 day, 5 day, and 9 month post‐surgery, detected in the patient's peripheral blood serum by flow cytometry and meso scale discovery technique

	Pre‐surgery	Day 1	Day 5	9 months
Lymphocyte subpopulations (cells/nL)				
B cells	94	162.4	61.5	105
NK cells	170.3	120.8	112.5	219.9
T cells	622.66	768.7	457.26	770.54
CD4+ T cells	454.83	589.47	345.02	565.35
CD8+ T cells	145.99	155.5	98.32	179.05
CD4+ T cell subpopulations (cells/nL)				
CD4+ naive	120.08	168.34	106.4	153.49
CD4+ CM	304.61	393.77	217.25	385.61
CD4+ EM	29.53	27.06	21.06	25.74
CD8+ T cell subpopulations (cells/nL)				
CD8+ naive	14.46	18.67	8.98	18.95
CD8+ CM	29.25	37.06	18.13	40.45
CD8+ EM	30.97	32.1	29.29	37.33
CD8+ TEMRA	71.31	67.67	41.92	82.31
Cytokines (pg/mL)				
TNF‐α	2.65	3.98	3.77	3.15
IFN‐γ	4.39	4.02	7.59	5.29
IL‐4	0	0.05	0.01	0.01
IL‐10	0.19	1.21	0.3	0.34
IL‐6	0.79	139.97	5.58	1.76

Central memory (CM); effector memory (EM); interferon (IFN)‐γ; interleukin (IL); natural killer (NK); transforming growth factor (TGF)‐β; terminally differentiated effector memory cells re‐expressing CD45RA (TEMRA); tumour necrosis factor (TNF)‐α.

For TNF‐α, the serum concentration increased on day 1 compared with pre‐surgical values and steadily decreased afterwards until 9‐months values were similar to pre‐surgical values. Interestingly, IFN‐γ concentrations only increased on day 5 before also decreasing again to a pre‐surgical level at the 9‐months follow‐up. Overall concentration levels for IL‐4 and IL‐10 were low, yet both cytokines showed a notable increase on day 1 compared with pre‐surgical values before decreasing again on day 5 and remaining slightly elevated at the 9‐months follow‐up compared with pre‐surgical values. IL‐6 concentrations showed the most prominent increase on day 1 compared with all other cytokine measurements. IL‐6 values then dropped again on day 5 and remained slightly elevated at the 9‐months follow‐up compared with pre‐surgical values (*Figure* 5B). For numeric values, please see *Table* 1.

## Discussion

Chronic QTRs are a rare yet challenging disease entity to treat, due to the often degenerated and fibrotic tissue surrounding the ruptured ends. As evidenced by the current case, a sole surgical repair of the injury site may often not satisfy the complex demands of a chronically ruptured tendon reconstruction.

PLX‐PAD cell injections were recently shown to induce an early inflammatory response including locally elevated gene expression levels of IL‐1β and IL‐6, which effectively improved tendon mechanics in an experimental patella tendon degeneration rat model.^18^ The physiological early inflammatory response in tendon healing is dominated by an expression of pro‐inflammatory IL‐1β and VEGF,^19^ two signal proteins which were previously shown to be increased by PLX‐PAD cells *in vivo* ^18^ and *in vitro*.^17^

It can be stipulated that PLX‐PAD cells may enhance the naturally occurring early repair phase by some degree of immunomodulation, which was also shown by an enhanced production of anti‐inflammatory IL‐10 when PLX‐PAD cells were cocultured with lipopolysaccharide‐activated peripheral blood mononuclear cells.^20^

Whether the herein observed systemically elevated concentrations of TNF‐α, IL‐4, IL‐6, and IL‐10 on day 1 following surgery were due to the commonly observed post‐surgery inflammatory host response,^21^, ^22^ the anaesthesia,^23^ or whether the PLX‐PAD treatment influenced the enhanced systemic cytokine response remains speculative when studying an isolated case.

A gradual increase of local CD4+ effector T cells was recently discovered to occur after tendon injuries *in vivo*, coordinated by local lymph nodes as part of the adaptive immune cell response to injury.^24^ A drop in systemic CD4+ T cell concentrations is however expected to appear following surgical interventions as an operation‐associated stress reaction. PLX‐PAD cells were shown to counteract this phenomenon when injected intramuscularly for the treatment of iatrogenic muscle injury following hip arthroplasty.^16^ In accordance with these findings, we were able to see elevated CD4+ T cell levels following the surgical intervention in the current case.

The main role of the quadriceps muscle during gait is to support body weight during knee flexion. To date, there are no detailed data available on gait function following surgically or conservatively treated QTRs.^25^ Here, we showed that a pre‐operative side‐to‐side deficit during knee flexion was resolved following surgical reconstruction of the quadriceps tendon. Considering the critical role of the quadriceps tendon during knee extension,^26^ QTRs result in uncontrolled and excessive knee extension during non‐weight‐bearing swing phases^27^ and should be reconstructed to ensure early return to function. This aligns with our pre‐surgical observation of an increased ROM during maximal active knee flexion and extension, which was markedly reduced postoperatively.

To achieve favourable clinical outcomes and low complication rates following QTR, early surgical treatment, use of augmentation techniques and a strict postoperative immobilization regimen are discussed as relevant measures to implement perioperatively.^28^ We followed these suggestions in addition to the compassionate use of intramuscular and intratendineous PLX‐PAD cell injections.

Because of the nature of this case, it must be discussed whether the successful outcome was achieved by the surgical approach, the PLX‐PAD cell injections or a combination of both. As previous operations were not conducted at our centre, we cannot be certain about the exact surgical interventions, however, it was confirmed that the patient was previously treated with biodegradable suture anchors. Although we used titanium suture anchors, studies show no differences between the two materials regarding dislocation rates and functional outcomes.^29^, ^30^, ^31^ The previous operations were conducted without the use of synthetic mesh augmentations, allografts or autografts. Because of the significant retraction distance, we decided to use a synthetic mesh in order to augment the tendon and superior capsule and to serve as a soft tissue cover.

As there is currently no standardized strategy available for the treatment of chronic QTRs, surgical algorithms are merely based on personal experience and case reports.^6^, ^7^, ^8^, ^9^, ^10^, ^11^, ^12^, ^13^ In 2015, Rehman and Kovacs described a case in which a chronic QTR was treated successfully by a combined hamstring autograft and Prolene® mesh augmentation and an injection of autologous platelet‐rich plasma (PRP) to enhance graft incorporation.^32^ Similar to PRP, PLX‐PAD cells have rich immunomodulatory and angiogenic capacities, which have previously been shown to increase muscle volume and strength after iatrogenic muscle injury^16^ and to improve blood flow in critical limb ischemia and peripheral artery disease.^15^, ^33^, ^34^

Although this report only covers an isolated case, our findings demonstrate a promising complementary treatment opportunity by combining a surgical‐mechanical enforcement strategy with a local immunomodulatory cell‐based therapy as a salvage treatment option for chronic QTRs. This approach is highly personalized and may only be considered for cases of chronic QTRs that are at high risk of treatment failure. High risk groups include patients with a history of long‐term steroid intake, multiple previous revision surgeries, and superficial or deep wound infections.

As we strive to further explore regenerative cell‐based treatments for musculoskeletal diseases, this mechano‐mechanistical approach warrants specific attention. A clinical trial to expand our knowledge regarding the multitudinous and complex interplay of injured tendinous tissue and PLX‐PAD cells is currently underway.

## Conflict of interest

R.O. is an employee and shareholder of Pluristem Ltd. P.R., C.P., and G.N.D. received institutional support from Pluristem Ltd. T.W. is a member of the clinical advisory board of Pluristem Ltd. for future indications and in the past received consulting fees from Pluristem Ltd., but not for this project. T.W. filed a patent together with Pluristem Ltd.

## Funding

No funding was received for the current case report.

## Supporting information


**Data S1.** Supporting information.

**Data S2.** Supporting information.
