# Supplementary material for: Intramuscular and intratendinous placenta‐derived mesenchymal stromal‐like cell treatment of a chronic quadriceps tendon rupture
Source: J Cachexia Sarcopenia Muscle. 2022 Jan 5;13(1):434–42. doi: 10.1002/jcsm.12894 (PMC8818634; doi:10.1002/jcsm.12894)
Supplement: Supplementary file 1 — Data S1. Supporting information. [file JCSM-13-434-s001.docx]

**Supporting Information S1, Gait analyses:** At each follow-up, the patient donned spandex shorts and his own walking shoes prior to the affixation of 60 reflective markers on relevant anatomical landmarks as suggested for the OCST, SARA and SCoRE combined approach (OSSCA) (1). Kinematic data of the lower extremities were collected using ten infrared cameras (*f*=120 Hz, MX-T20, VICON, Oxford, UK) while the patient walked at a self-selected speed along a 10-m marked pathway, in which two embedded force platforms (*f*=960 Hz, AMTI-BP400600, Watertown, MA, USA) simultaneously collected ground reaction forces (GRF). Walking trials were included for analysis if both feet contacted the force platforms cleanly; a minimum of 5 valid trials per visit were collected. Two elbow crutches were used as walking aids at both pre-operative and 3-months post-operative sessions. At the 9-months follow-up, the patient walked independently. In addition to gait analysis, the active knee ROM was evaluated separately on each limb, where a series of 5 voluntary, maximal flexion-extension swings was performed by the patient in a one-legged standing position. Further, knee extensor muscle strength was assessed on each limb with hand-held dynamometry (MicroFET2, Hogan Health Industries, Inc., West Jordan, UT, USA).

After post-processing (VICON Nexus 1.8.2, Oxford, UK), spatiotemporal gait parameters were determined from walking trial data. All further calculations were performed using custom MATLAB scripts (R2019b, MathWorks, Natick, MA, USA). For both active knee ROM and walking trials, sagittal knee angles were calculated by implementing the OSSCA projection, from which the overall ROM was determined. Sagittal knee angles from walking trials were divided into gait cycles for further analysis. Stance phases were extracted from GRF data using a 25 N vertical GRF threshold. Mean curves for both knee angles and GRF were computed using a dynamic time warping procedure (2). Fig.4 was created with GraphPad Prism 9.

**References**

1. Taylor WR, Kornaropoulos EI, Duda GN, Kratzenstein S, Ehrig RM, Arampatzis A, et al. Repeatability and reproducibility of OSSCA, a functional approach for assessing the kinematics of the lower limb. Gait Posture. 2010;32(2):231-6.

2. Bender A, Bergmann G. Determination of typical patterns from strongly varying signals. Comput Methods Biomech Biomed Engin. 2012;15(7):761-9.
