# Supplementary material for: Intramuscular and intratendinous placenta‐derived mesenchymal stromal‐like cell treatment of a chronic quadriceps tendon rupture
Source: J Cachexia Sarcopenia Muscle. 2022 Jan 5;13(1):434–42. doi: 10.1002/jcsm.12894 (PMC8818634; doi:10.1002/jcsm.12894)
Supplement: Supplementary file 2 — Data S2. Supporting information. [file JCSM-13-434-s002.docx]

**Supporting Information S2, Biomarkers:** Blood samples were obtained from the patient through a peripheral venous puncture of the cubital vein after thorough disinfection of the puncture site. Blood was then centrifuged at 1200 g for 10 mins. After successful separation of serum and cell pallet, serum was pipetted off for flow cytometry panels for T and B cells and granulocytes. All samples were measured using a Navios flow cytometer (Beckman Coulter, Brea, CA, USA) and data was analyzed using the Kaluza software (Beckman Coulter). The Meso Scale Discovery® (Meso Scale Diagnostics, Rockville, MD, USA) technology was used to measure endogenous plasma levels with a panel of five soluble proinflammatory factors (TNF-α, IFN-γ, IL-4, IL-10, IL-6). Fig.5 was created with GraphPad Prism 9.
